# Regulation of laryngeal squamous cell cancer progression by the lncRNA H19/miR-148a-3p/DNMT1 axis

**DOI:** 10.18632/oncotarget.7270

**Published:** 2016-02-08

**Authors:** Tianyi Wu, Lingmei Qu, Guoqing He, Linli Tian, Liang Li, Han Zhou, Qian Jin, Jingyuan Ren, Yu Wang, Jingting Wang, Xuan Kan, Ming Liu, Jia Shen, Mian Guo, Yanan Sun

**Affiliations:** ^1^ Department of Otorhinolaryngology, Head and Neck Surgery, The Second Affiliated Hospital of Harbin Medical University, Harbin, China; ^2^ Department of Otorhinolaryngology, Head and Neck Surgery, The Fifth Affiliated Hospital of Harbin Medical University, Daqing, China; ^3^ Department of Head and Neck Surgery, The Oncology Hospital of Jilin province, Changchun, China; ^4^ Department of Orthopaedic Surgery and the Orthopaedic Hospital Research Center, University of California, Los Angeles, California, USA; ^5^ Department of Neurosurgery, The Second Affiliated Hospital of Harbin Medical University, Harbin, China

**Keywords:** laryngeal squamous cell cancer, lncRNA H19, miR-148a-3p, DNMT1

## Abstract

Laryngeal squamous cell carcinoma (LSCC) is a highly aggressive malignant cancer. The regulation of LSCC progression by long non-coding RNA (lncRNA) was not well understood. In this study, we reported that the lncRNA H19 was upregulated in LSCC. The expression levels of H19 were inversely correlated with the survival rate of LSCC patients. Knockdown of H19 expression inhibited LSCC cell migration, invasion and proliferation. We identified microRNA miR-148a-3p as an inhibitory target for H19. Overexpression of miR-148a-3p reduced LSCC migration, invasion and proliferation cell, while inhibition of miR-148a-3p did the opposite. The inhibition of LSCC progression induced by H19 knockdown required the activity of miR-148a-3p. We also identified DNA methyltransferase enzyme DNMT1 as a target of miR-148a-3p. Cellular DNA methylation levels were inhibited by both miR-148a-3p overexpression and H19 knockdown. In summary, our study demonstrated that the lncRNA H19 promoted LSCC progression via miR-148a-3p and DNMT1.

## INTRODUCTION

Head and neck squamous cell carcinoma is the sixth most common cancer in the world, among which the laryngeal squamous cell carcinoma (LSCC) is a highly aggressive malignancy [1]. Although encouraging progress in the diagnosis and treatment for LSCC has been achieved in the past 20 years, the overall survival rate remains unfavorable. A recent study has shown that the overall 1-and 2-year survival rates for LSCC patients without treatment are only 56.4% and 26.5%, respectively [2]. Recurrence and metastasis are believed to be the major factors that to limit the successful treatment of LSCC [3]. In order to develop effective therapy for LSCC, the efforts towards understanding the underlying pathological mechanisms of LSCC have been intensified recently.

Noncoding RNAs are subdivided into small ncRNAs (< 200 nt) and long ncRNAs (> 200 nt) based on their size. Small ncRNAs have been shown to act primarily as negative regulators of gene expression. A number of microRNAs, which belong to the small ncRNA family, have been demonstrated to function as oncogenes or tumor suppressor genes for LSCC in our previous studies [4–8]. Long ncRNAs (lncRNAs) are poorly conserved among species [9, 10], but accumulating evidences indicate that lncRNAs could play important roles in a variety of biological processes and may well be also involved in the development of cancer and other human diseases [11, 12]. Specifically, our previous studies have suggested the involvement of lncRNA HOTAIR in LSCC [13, 14]. The overall pathophysiological contribution of lncRNAs to LSCC, however, is largely unknown.

The lncRNA H19 is transcribed from a maternally expressed imprinted gene locus on human chromosome 11. The H19 gene encodes a 2, 600 nt capped, spliced, and polyadenylated noncoding RNA that is predominantly cytoplasmic [15, 16]. Although H19 has been intensively studied in the field of genomic imprinting, the biological function of H19 as a non-coding RNA has only recently begun to be elucidated. Accumulating evidence in recent studies has consistently demonstrated that the expression levels of H19 are upregulated in a variety of cancer types, including gastric cancer [17], esophageal cancer [18] colorectal cancer [19–21], breast cancer [22, 23], bladder cancer [24, 25], and hepatocellular carcinoma [26, 27]. The upregulation of H19 in cancer tissues suggests its possible tumorigenic properties, although the detailed molecular mechanism remains to be investigated.

In the present study, we found that the lncRNA H19 was upregulated in LSCC and that the expression levels of H19 were inversely correlated with the survival rate of LSCC patients. Consistently, we found that knockdown of H19 expression inhibited LSCC cell proliferation, migration and invasion. We identified microRNA miR-148a-3p as a target for H19. The expression of miR-148a-3p was inhibited by H19, and the overexpression and the inhibition of miR-148a-3p were respectively associated with reduced and elevated LSCC proliferation, migration and invasion. Importantly, the inhibition of LSCC progression induced by H19 knockdown required the activity of miR-148a-3p. We also determined that DNA methyltransferase enzyme DNMT1 as an inhibitory target of miR-148a-3p. Cellular DNA methylation was inhibited by both miR-148a-3p overexpression and H19 knockdown. Taken together, our study demonstrated that the lncRNA H19 could promote LSCC progression via miR-148a-3p and DNMT1, and that DNA methylation was involved in the regulatory mechanism.

## RESULTS

### H19 was upregulated in LSCC and was inversely correlated with patient survival rate

**Figure 1:**
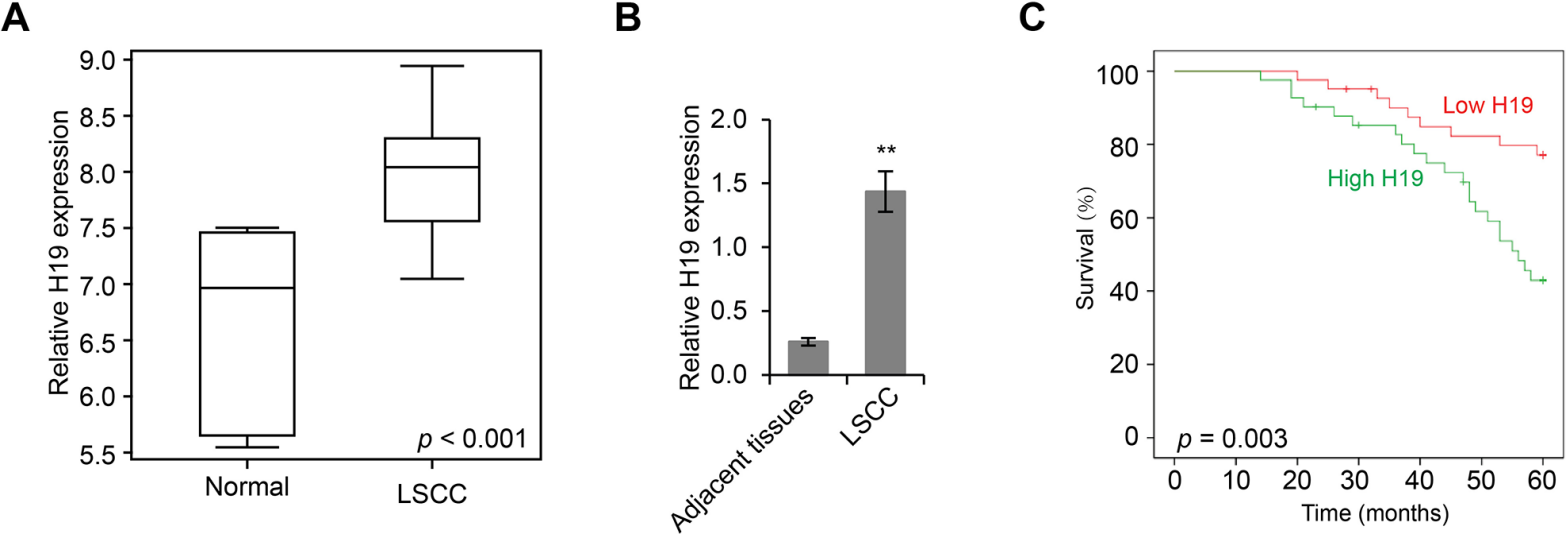
H19 is upregulated in LSCC and is inversely correlated with patient survival rate (**A**) Box plot of H19 expression levels in LSCC tissues and adjacent non-neoplastic normal tissues as determined by lncRNA-specific microarray analysis (*p* < 0.001). (**B**) Real time PCR analysis of H19 expression levels in LSCC tissues and adjacent non-neoplastic normal tissues (**p < 0.01). (**C**) The Kaplan-Meier overall survival rate curve for LSCC patients (*n* = 82) with high and low H19 expression levels (*p* = 0.003).

Since lncRNAs have been implicated in the development of cancers, we first sought to examine the landscape of lncRNA gene expression in LSCC with a hybridization microarray designed for lncRNAs. We found that the expression levels of H19 were significantly upregulated in LSCC tissue samples compared to those in normal tissues (*p* < 0.001, Figure 1A). With primers specific to H19, we validated our findings by qPCR analysis, and found that H19 levels were significantly higher (5.54-fold) in LSCC tumor tissues than those in adjacent non-neoplastic tissues (3.342 ± 1.436 versus 0.596 ± 0.259) (*p* < 0.01, Figure 1B). Furthermore, we determined that the expression levels of H19 were significantly correlated with the progression of LSCC, including tumor grade, differentiation, neck nodal metastasis, and clinical stage (Table 1). Based on the levels of H19 expression, we categorized 82 LSCC patients into high (*n* = 41) and low (*n* = 41) H19 expression groups. With Kaplan-Meier analysis, we found that patients with high H19 expression had significantly poorer overall survival rate compared to those with low H19 expression (χ^2^ = 8.704, *p* = 0.003) (Figure 1C). Taken together, these results indicated that H19 was upregulated in LSCC and was positively correlated with LSCC progression.

**Table 1:** Relationship between H19 expression level and clinicopathologic parameters of LSCC

Characteristics (*n*)	H19 level	*P*
**Sex**		0.638
Male (55)	3.295 ± 1.580	
Female (27)	3.440 ± 1.142	
**Age**		0.122
≥ 58 (41)	3.590 ± 1.426	
< 58 (41)	3.095 ± 1.438	
**T classification**		< 0.01
T1–2 (49)	2.890 ± 1.333	
T3–4 (33)	4.014 ± 1.357	
**Differentiation**		0.017
G1 (58)	3.068 1.332	
G2 (24)	3.993 1.553	
**Lymph node metastasis**		< 0.01
Negative (52)	2.825 ± 1.281	
Positive (30)	4.240 ± 1.278	
**Primary location**		0.103
Supraglottic (35)	3.639 ± 1.357	
Glottic (47)	3.121 ± 1.482	
**Clinical stage**		< 0.01
I–II (45)	2.867 ± 1.356	
III–IV (37)	3.92 ± 1.351	

### H19 knockdown inhibited LSCC cell migration, invasion and proliferation

**Figure 2:**
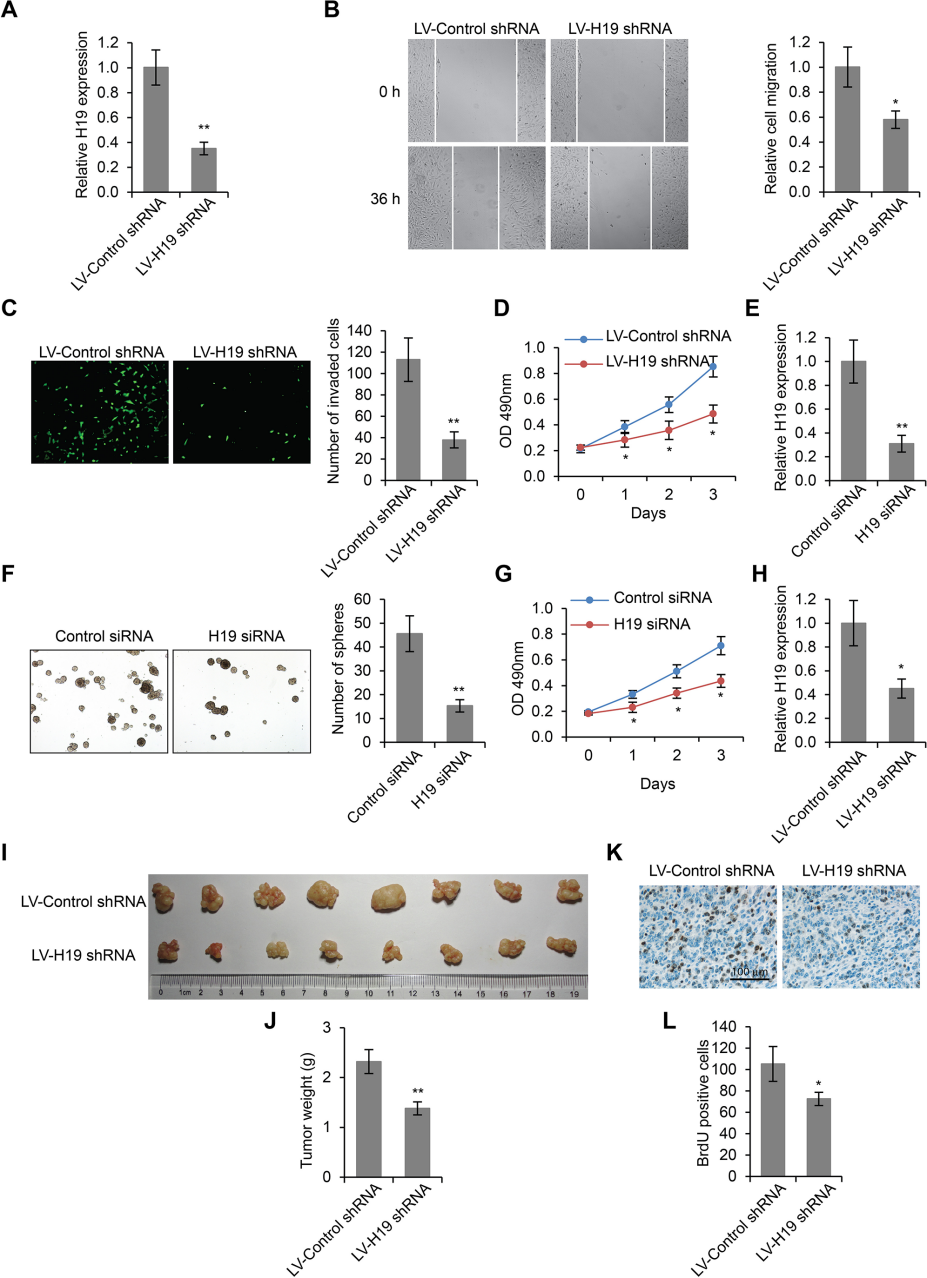
H19 knockdown inhibits LSCC cell migration, invasion and proliferation (**A**) H19 expression levels in Hep-2 cells lentiviruses encoding control shRNA or H19 shRNA. (**B**) Wound healing cell migration assay, (**C**) Transwell cell invasion assay and (**D**) MTS cell proliferation assay in Hep-2 cells transfected with lentiviruses encoding control shRNA or H19 shRNA. (**E**) H19 expression levels in LSCC-SCs transiently transfected with control siRNA or H19 siRNA. (**F**) Sphere formation in LSCC-SCs transfected with control siRNA or H19 siRNA. (**G**) MTS cell proliferation assay in LSCC-SCs transfected with control siRNA or H19 siRNA. (**H**) H19 expression levels in subcutaneous xenograft LSCC tumors transfected with lentiviruses encoding control shRNA or H19 shRNA. (**I**) Subcutaneous xenograft LSCC tumors developed in nude mice from Hep-2 cells transfected with lentiviruses encoding control shRNA or H19 shRNA. (**J**) Weight quantification of tumor tissues depicted in (I). (**K**) Immunohistochemistry staining of BrdU in tumor tissues depicted in (I). Scale bar = 100 μm. (**L**) Quantification of BrdU positive cells. *p < 0.05 and **p < 0.01 compared to the control group.

In order to investigate the function of H19 in LSCC development, we knocked down the expression of H19 by transfecting lentiviruses encoding control shRNA or H19 shRNA into Hep-2 cells, a well-established LSCC cell line. The H19 expression levels were significantly reduced by this treatment after 24 h (*p* < 0.01, Figure 2A). We then performed wound healing cell migration assay on these cells. We found that the migration of Hep-2 cells was significantly inhibited by H19 knockdown (*p* < 0.05, Figure 2B). We also performed transwell assay to examine cell invasion ability, and found that compared to shRNA control, the shRNA targeting H19 led to significantly decreased number of transmembrane cells (*p* < 0.01, Figure 2C). Moreover, by MTS assay, we discovered that H19 knockdown also significantly inhibited cell proliferation (*p* < 0.05, Figure 2D). Furthermore, we generated LSCC stem cells (LSCC-SCs) from LSCC patient and knocked down H19 expression (Figure 2E). These LSCC-SCs were subjected to sphere formation and MTS assays to examine whether H19 influences LSCC-SC proliferation. The results revealed that downregulated of H19 significantly suppressed LSCC-SC growth (Figure 2F and 2G). Taken together, decreased H19 expression led to impaired cell migration, invasion and proliferation in LSCC cells.

In addition to *in vitro* experiments, we used a mouse xenograft model to study the oncogenic role of H19 in LSCC development *in vivo*. All mice subcutaneously injected with Hep-2 cells developed detectable tumors in our study. We determined that the tumor growth was significantly inhibited in mice treated with lentivirus encoding H19 shRNA (Figure 2H), compared to those treated with lentivirus encoding control shRNA, evidenced by significantly reduced weight of tumor mass (1.272 ± 0.213g vs 2.112 ± 0.273g, *p* < 0.01, Figure 2I and 2J). BrdU staining of the tumor tissue revealed reduced cell proliferation in H19 shRNA treated mice (*p* < 0.01, Figure 2K and 2L), further confirming the stimulatory role of H19 during LSCC development.

### MiR-148a-3p, an inhibitor of LSCC development, was an inhibitory target of H19

**Figure 3:**
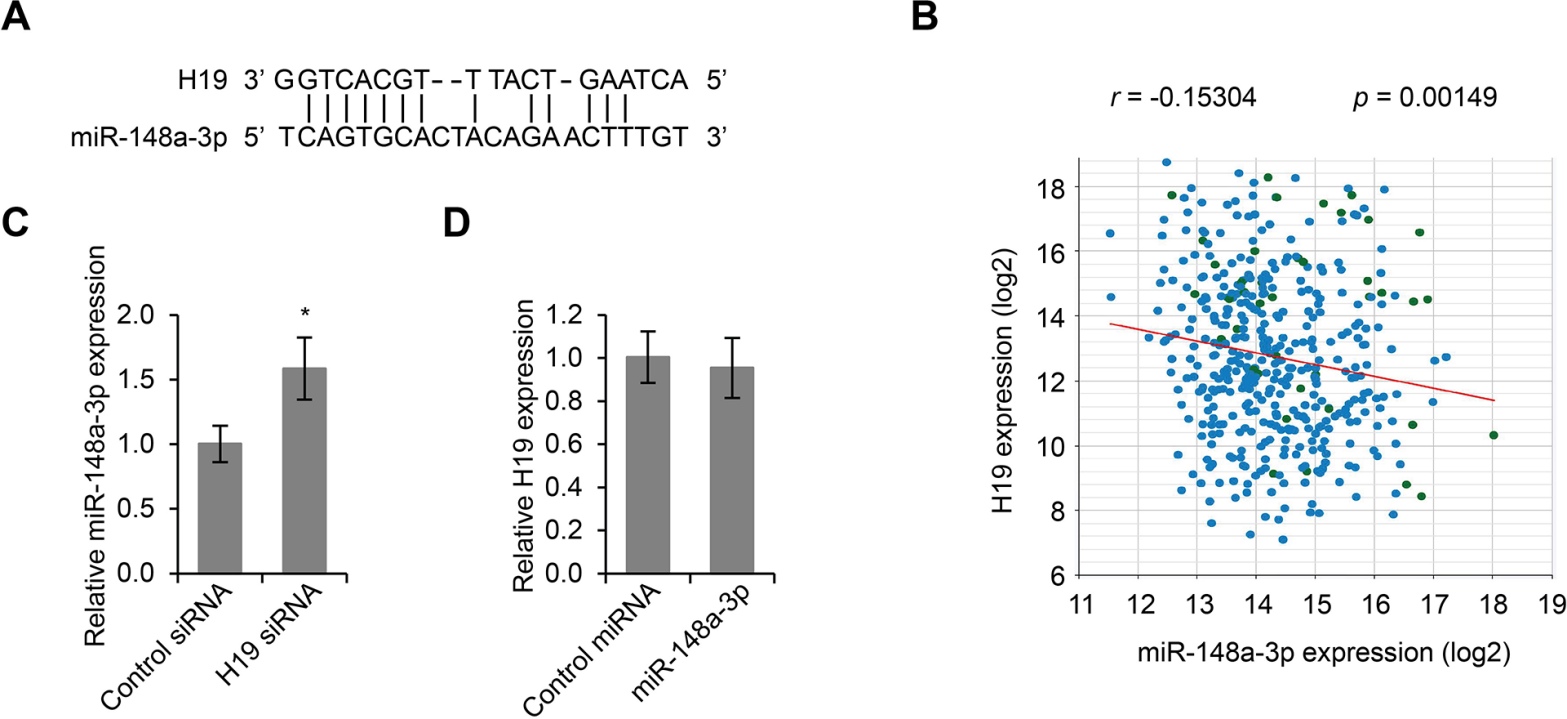
MiR-148a-3p is an inhibitory targeted of H19 (**A**) Sequence complementarity between H19 and miR-148a-3p. (**B**) Pearson correlation of H19 and miR-148a-3p expression in LSCC tissues (*r* = −0.15304, *p* = 0.00149). (**C**) Real time PCR analysis of miR-148a-3p expression in Hep-2 cells transfected with control siRNA or H19 siRNA. *p < 0.05 compared to control group. (**D**) Real time PCR analysis of H19 expression in Hep-2 cells transfected with control miRNA or miR-148a-3p.

In order to investigate the molecular mechanism by which H19 regulates LSCC development, we first utilized starBase v2.0 to predict H19 targets based on sequence complementarity. A microRNA, miR-148a-3p, which had 13 base pair match with H19, became a potential candidate (Figure 3A). We further extracted LSCC gene expression data from public databases, and performed Pearson correlation analysis, and found that the expression levels of miR-148a-3p and H19 were negatively correlated (*r* = −0.15304, *p* = 0.00149, Figure 3B). In addition, this prediction was then confirmed by qPCR assay using primers specific to miR-148a. Reduction of H19 expression by siRNA led to significantly increased expression levels of miR-148a-3p, indicating that miR-148a-3p was an inhibitory target for H19 (*p* < 0.05, Figure 3C). Importantly, overexpression of miR-148a-3p was not able to affect the expression levels of H19 (Figure 3D), further confirming that H19 was upstream of miR-148a-3p.

**Figure 4:**
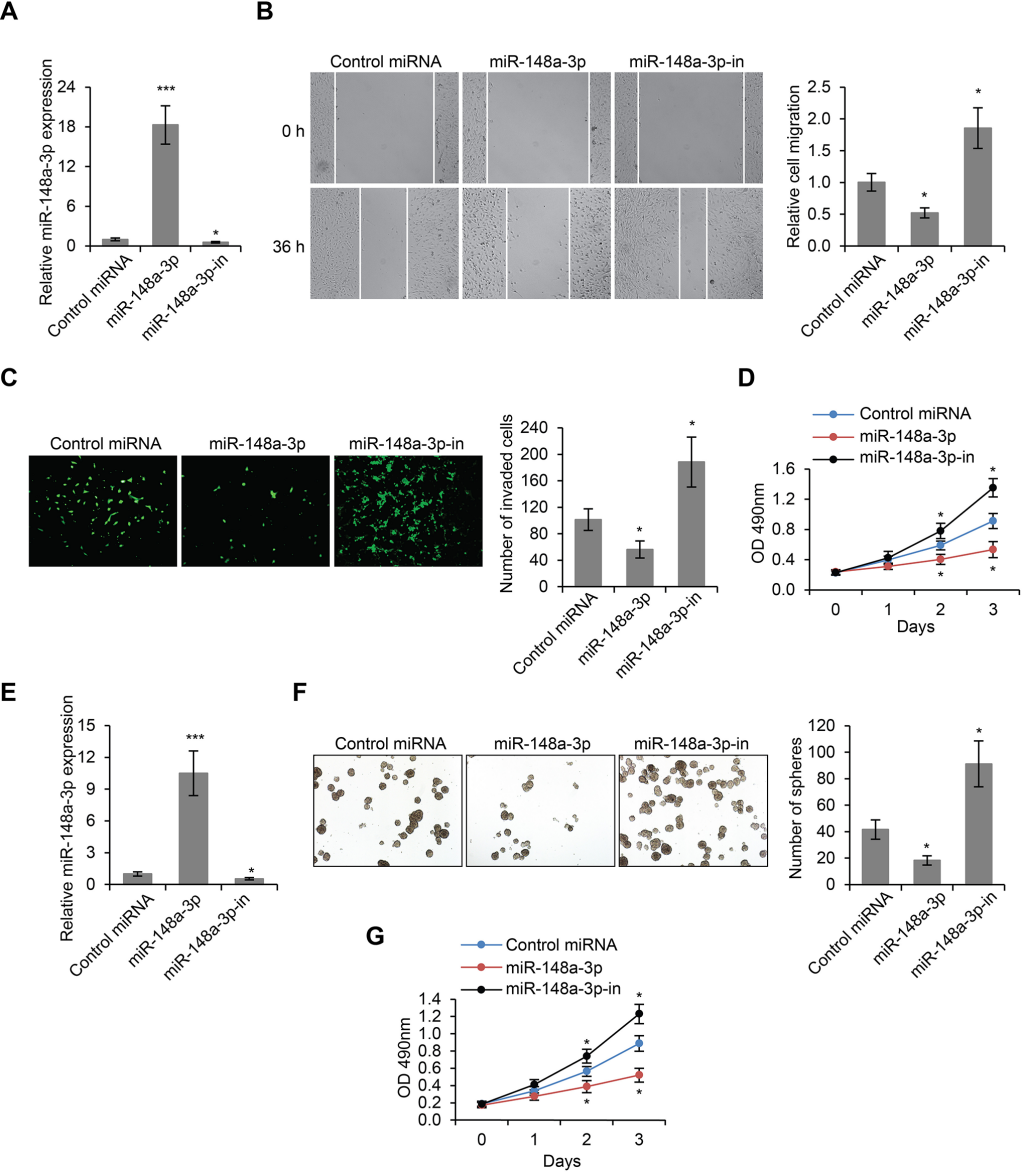
MiR-148a-3p is an inhibitor of LSCC development (**A**) miR-148a-3p expression levels as determined by real time PCR in Hep-2 cells transfected with control miRNA, miR-148a-3p or miR-148a-3p inhibitory oligo (miR-148a-3p-in). (**B**) Wound healing cell migration assay, (**C**) Transwell cell invasion assay and (**D**) MTS cell proliferation assay in Hep-2 cells transfected with control miRNA, miR-148a-3p or miR-148a-3p-in. (**E**) miR-148a-3p expression levels in LSCC-SCs transfected with control miRNA, miR-148a-3p or miR-148a-3p-in. (**F**) Sphere formation assay in LSCC-SCs transfected with control miRNA, miR-148a-3p or miR-148a-3p-in. (**G**) MTS cell proliferation assay in LSCC-SCs transfected with control miRNA, miR-148a-3p or miR-148a-3p-in. *p < 0.05, ***p < 0.001 compared to the control group.

In order to investigate the role of miR-148a-3p in LSCC tumorigenesis, we modulated the expression of miR-148a-3p in Hep-2 cells by transfecting vectors overexpressing miR-148a-3p, or vectors expressing an inhibitory sequence of miR-148a-3p (miR-148a-3p-in) (Figure 4A). We then performed wound healing assay on these transfected cells. We found that the overexpression of miR-148a-3p led to significantly reduced cell migration, and that inhibition of miR-148a-3p expression significantly promoted cell migration (*p* < 0.01, Figure 4B). Furthermore, transwell cell invasion assays revealed that the invasive abilities of Hep-2 cells were remarkably reduced by miR-148a-3p overexpression, and were significantly enhanced by miR-148a-3p inhibition (*p* < 0.01, Figure 4C). Additionally, we found that the overexpression of miR-148a-3p also inhibited cell proliferation, whereas the inhibition of miR-148a-3p expression significantly increased the proliferation of Hep-2 cells (*p* < 0.01, Figure 4D). These results were further confirmed by LSCC stem cells. We transfected control miRNA, miR-148a-3p or miR-148–3p-in into LSCC-SCs (Figure 4E) and then performed sphere formation and MTS assays. We found that miR-148a-3p transfection inhibited cell proliferation, while miR-148a-3p-in transfection stimulated cell proliferation (Figure 4F and 4G). Taken together, miR-148a-3p played a negative role on cell migration, invasion and proliferation, which was consistent with its role as an inhibitory target of H19.

### DNMT1 was a target gene of miR-148a-3p and was regulated by H19

**Figure 5:**
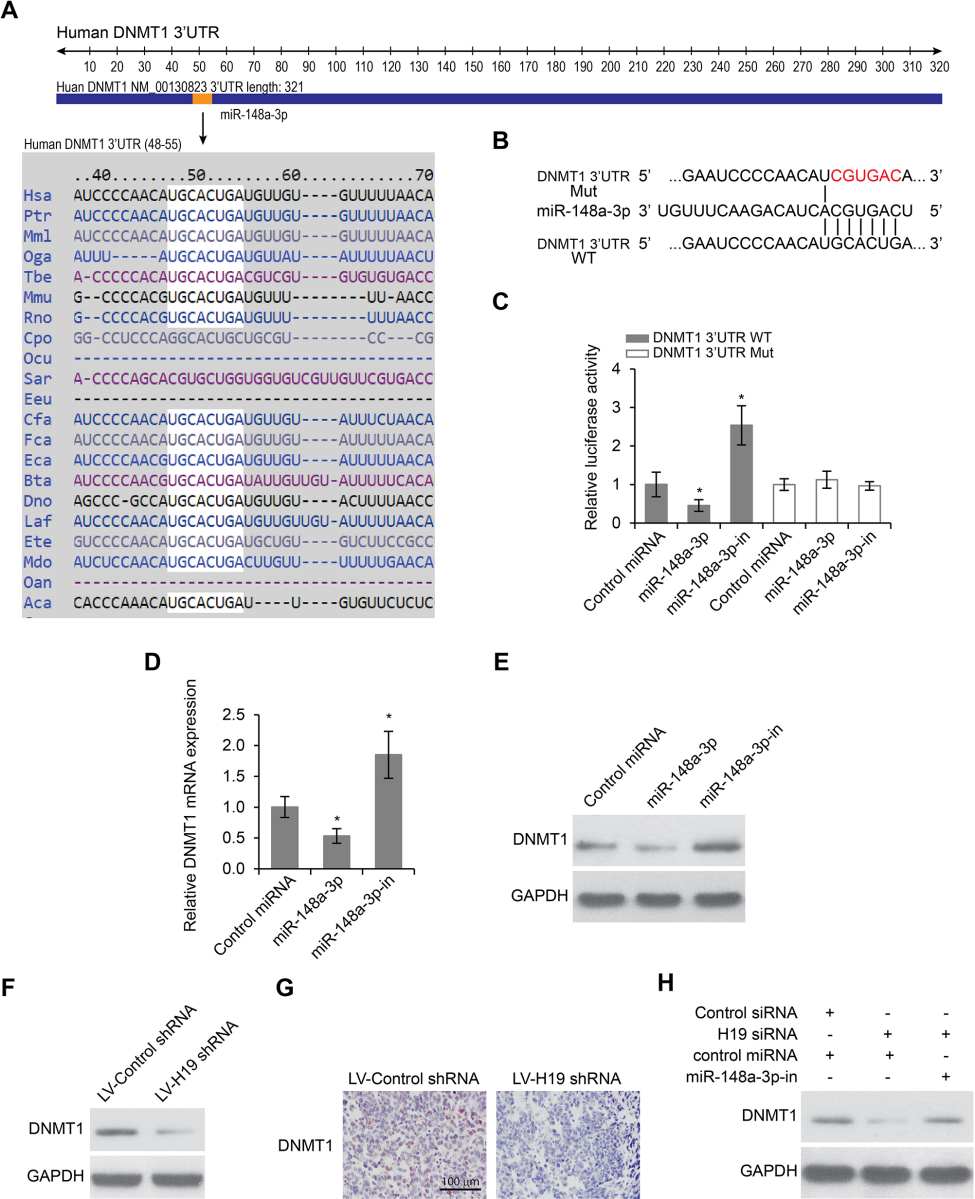
DNMT1 is a target of miR-148a-3p and is suppressed by H19 deletion (**A**) Prediction of DNMT1 as a target of miR-148a-3p in different species. (**B**) Schematic view of miR-148a-3p putative targeting site in the wild type (WT) and mutant (Mut) 3′-UTR of DNMT1. (**C**) Luciferase activity assay in Hep-2 cells transfected with luciferase report plasmids containing DNMT1 3′UTR (WT or Mut) with control miRNA, miR-148a-3p or miR-148a-3p-in. (**D**) Relative mRNA and (**E**) Relative protein levels of DNMT1 in Hep-2 cells transfected with control miRNA, miR-148a-3p or miR-148a-3p-in. *p < 0.05 compared to the control group. (**F**) Protein levels of DNMT1 in Hep-2 cells transfected with lentiviruses encoding control shRNA or H19 shRNA as determined by Western blot analysis. (**G**) Immunohistochemistry staining of DNMT1 in xenograft LSCC tumors developed in nude mice from Hep-2 cells transfected with lentiviruses encoding control shRNA or H19 shRNA. Scale bar = 100 μm. (**H**) Protein levels of DNMT1 expression in Hep-2 cells transfected with control siRNA or H19 siRNA in the presence or absence of miR-148a-3p-in.

We used TargetScan and miRanda to predict target genes for miR-148a-3p, and DNMT1 came out as one of the best candidates (Figure 5A). In order to confirm the prediction, we first constructed luciferase reporter plasmids harboring either the wild-type 3′-UTR of DNMT1 or a mutant 3′-UTR predicted to be insensitive to miR-148a-3p (Figure 5B). We then transfected Hep-2 cells with the luciferase reporter plasmids, together with plasmids either overexpressing or inhibiting miR-148a-3p. We found that miR-148a-3p overexpression significantly reduced the luciferase activity driven by wild-type 3′-UTR of DNMT1, while miR-148a-3p inhibition significantly increased the luciferase activity. The regulation mechanism was dependent on the recognition between miR-148a-3p and the 3′-UTR of DNMT1, because neither overexpression nor inhibition of miR-148a-3p were able to change the luciferase activity driven by mutant 3′-UTR of DNMT1 that was insensitive to miR-148a-3p (Figure 5C). To further confirm the regulation of DNMT1 by miR-148a-3p, we examined both the mRNA and the protein levels of DNMT1 when miR-148a-3p was overexpressed or inhibited. We found that the overexpression of miR-148a-3p significantly decreased both the mRNA and the protein levels of DNMT1, whereas the inhibition of miR-148a-3p significantly increased both the mRNA and the protein levels of DNMT1 (Figure 5D and 5E). Taken together, these results indicated that DNMT1 was a target gene of miR-148–3p.

Since DNMT1 was a target of miR-148a-3p, and miR-148a-3p itself is a target of H19, we wanted to determine whether or not the expression of DNMT1 was regulated by H19. In Hep-2 cells, we found that knockdown of H19 led to decreased protein levels of DNMT1 (Figure 5F). In xenograft LSCC tissues from mice treated with lentivirus encoding H19 shRNA, the protein levels of DNMT1 were also reduced by immunohistochemistry staining, compared to LSCC tissues from mice treated with a control lentivirus (Figure 5G). Significantly, the regulation of DNMT1 by H19 required the activity of miR-148a-3p, because we found that knockdown of H19 was no longer able to reduce DNMT1 expression when miR-148a-3p was inhibited (Figure 5H). These data together indicated that H19 positively regulated the expression of DNMT1 via inhibiting miR-148a-3p.

### The regulation of LSCC cell migration, invasion and proliferation by H19 was dependent on miR-148a-3p, and was associated with DNA methylation

**Figure 6:**
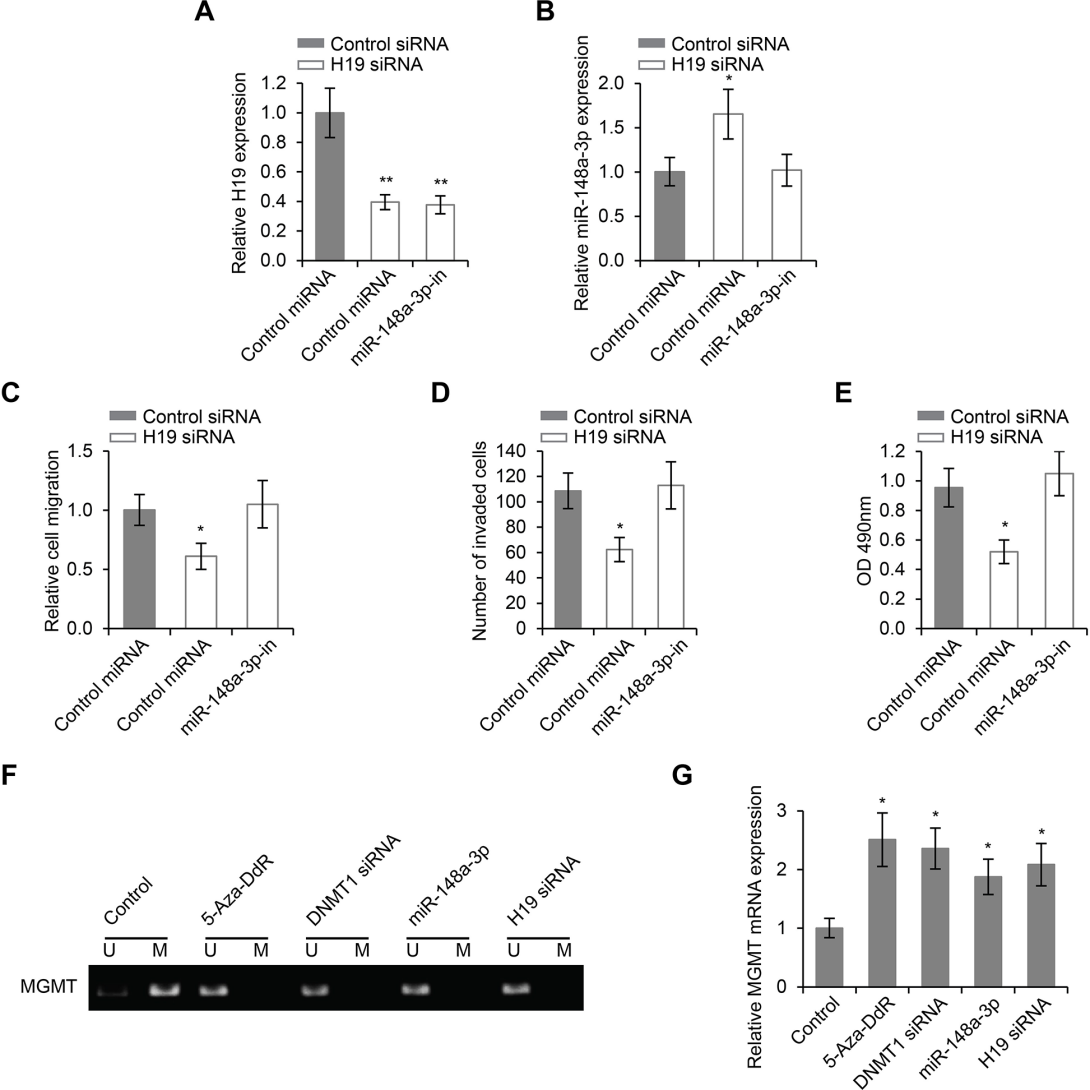
The regulation of LSCC progression by H19 is dependent on miR-148a-3p, and is associated with DNA methylation (**A**) Relative expression levels of H19 and (**B**) Relative expression levels of miR-148a-3p in Hep-2 cells transfected with control siRNA or H19 siRNA in the presence or absence of miR-148a-3p-in. (**C**) Wound healing cell migration assay, (**D**) Transwell cell invasion assay and (**E**) MTS cell proliferation assay in Hep-2 cells transfected with control siRNA or H19 siRNA in the presence or absence of miR-148a-3p-in. (**F**) The methylation status of MGMT in Hep-2 cells treated with 5-Aza-DdR, DNMT1 siRNA, miR-148–5a or H19 siRNA (U: methylated alleles; M: unmethylated alleles). (**G**) The expression levels of MGMT mRNA in Hep-2 cells treated with 5-Aza-DdR, DNMT1 siRNA, miR-148–5a or H19 siRNA. *p < 0.05, **p < 0.01 compared to the control group.

Previously in this study, we demonstrated that H19 knockdown inhibited LSCC cell migration, invasion and proliferation. Given the findings that miR-148a-3p was a target of H19, we sought to determine whether or not the regulation of LSCC cell migration, invasion and proliferation by H19 was dependent on miR-148a-3p. As expected, inhibition of miR-148a-3p did not change the expression levels of H19 (Figure 6A), but decreased the expression levels of miR-148a-3p induced by H19 knockdown (Figure 6B). Inhibition of miR-148a-3p completely abrogated the inhibitory effects on cell migration, invasion and proliferation caused by H19 knockdown (Figure 6C, 6D, and 6E), suggesting that the stimulatory roles of H19 on LSCC cell migration, were dependent on the activity of miR-148a-3p.

In order to explore the roles of DNMT1 in LSCC progression, we focused on the regulation of gene promoter methylation by both H19 and miR-148a-3p, since DNMT1 was known to participate in this biological function [28]. We first examined the global methylation of gene promoters by MeDIP and microarray hybridization. We found that there were more promoter hypomethylation than hypermethylation in cells where H19 was knocked down (Table 2). In addition, both miR-148–3p overexpression and H19 knockdown inhibited promoter methylation of the MGMT gene, a phenotype also observed with DNMT1 knockdown and with the treatment of DNA methylation inhibitor, 5-Aza-DdR (Figure 6F). The expression of MGMT was induced when its promoter was methylated, and this induction was achieved by DNMT1 knockdown, miR-148–3p overexpression and H19 knockdown (Figure 6G). These results collectively suggested that DNA methylation was involved in the regulation of LSCC progression by the H19/miR-148a-3p/DNMT1 cascade.

**Table 2:** The methylation status of Hep-2 cell after H19 downregulation compared with the control Hep-2 cells

Chromosome	Hypermethylation (gene number)	Hypomethylation (gene number)
chr1	209	−272
chr2	113	−189
chr3	127	−154
chr4	73	−105
chr5	61	−143
chr6	102	−157
chr7	75	−120
chr8	64	−96
chr9	61	−120
chr10	78	−122
chr11	116	−177
chr12	94	−149
chr13	28	−56
chr14	57	−103
chr15	70	−113
chr16	70	−128
chr17	109	−156
chr17_random	0	−1
chr18	21	−33
chr19	109	−167
chr20	48	−85
chr17_random	0	−1
chr18	21	−33
chr19	109	−167
chr20	48	−85
chr21	21	−30
chr22	52	−68
chrX	79	−110
chrY	10	−26

## DISCUSSION

The development of LSCC involves various genetic and epigenetic machineries, including the three susceptibility loci identified by our previous study [29]. With recent advancement of the knowledge of non-coding RNAs, a number of microRNAs and lncRNAs have been shown to participate in cancer development [30–32]. In this study, we first carried out a specifically designed microarray analysis comparing the expression profile of only lncRNAs in LSCC tissues and adjacent non-neoplastic normal tissues. Since the expression levels of most lncRNAs are much lower than protein coding genes, this approach allowed us to focus on the differential expression of lncRNAs, without the interfering background from protein coding mRNAs. We demonstrated that the lncRNA H19 was upregulated in LSCC, and that the expression levels of H19 were inversely correlated with the survival rate of LSCC patients. These findings strongly supported the hypothesis that H19 stimulated LSCC progression. Furthermore, loss of endogenous H19 exerted inhibitory effects on the proliferation and invasion of LSCC cells, suggesting that H19 was required for the development of LSCC.

To delineate the mechanism by which lncRNAs regulate gene expression has been very challenging. Several recent studies suggested that the regulatory mechanism encompassed almost every aspect of pre- and post-transcriptional processes, including transcription, post-transcriptional processing, chromatin modification, and the modulation of protein function [33–35]. In this context, although it appears that H19 consistently promotes the development of multiple types of cancers [17, 19–27], its mechanism of action is not understood. In this study, we identified microRNA miR-148a-3p as an inhibitory target of H19 by sequence complementarity analysis. It is not uncommon that a non-coding RNA is the target of another non-coding RNA. For example, miR-200, let-7 and miR-675 are demonstrated in previous studies to be regulated by H19 [36–39]. Importantly, not only could miR-148a-3p by itself regulate LSCC progression, the regulation of LSCC progression by H19 also required the activity of miR-148a-3p. Loss of miR-148a-3p almost completely reversed the phenotype induced by H19 knockdown. These results indicated that miR-148a-3p is downstream of H19 in a signaling cascade that regulated LSCC progression. Although based on the sequence complementarity between H19 and miR-148a-3p, it is reasonable to believe that miR-148a-3p is likely a direct target of H19, but because the interaction between RNA molecules could also be affected by other factors including the secondary structure, we therefore could not exclude the possibility that H19 targeted other miRNAs, which then served as an intermediate regulator of the expression of miR-148a-3p.

In this study, we also identified DNA methyltransferase enzyme DNMT1 as a target of miR-148a-3p. The expression levels of DNMT1, and the levels of cellular DNA methylation, especially the methylation of gene promoters, were regulated by both miR-148a-3p and H19. It has been reported by previous studies that non-coding RNAs were able to alter the epigenetic status of the cells, but the detailed mechanisms were not well understood. Our findings in this study have provided new insight into the mechanism by which non-coding RNAs modulate cellular epigenetic status. Taken together, our study demonstrated that the lncRNA H19 could promote LSCC progression via miR-148a-3p and DNMT1, and that DNA methylation was an important factor involved in the regulatory mechanism.

## MATERIALS AND METHODS

### Tissue sample

Included in the study were patients with laryngeal cancer who underwent partial or total laryngectomy at the Department of Otorhinolaryngology of the Second Affiliated Hospital of Harbin Medical University between October 2006 and July 2008. Five matched samples of LSCC tissues and the corresponding adjacent non-neoplastic tissues were tested by microarray. 82 matched cancerous and noncancerous tissues were tested by real-time qPCR. The study was approved by the Ethics Committee of Harbin Medical University and informed consent was obtained.

### RNA extraction, RNA labeling and array hybridization

The total RNA was isolated from laryngeal carcinoma and corresponding adjacent non-neoplastic tissues or Hep-2 cells using Trizol reagent (Invitrogen, Carlsbad, CA, USA) and quantified using the NanoDrop 1000 (NanoDrop Technologies, Rockland, DE, USA), and the RNA integrity was assessed using standard denaturing agarose gel electrophoresis. The Human 8 × 60 k long non-coding RNA array was manufactured by Arraystar Company (Rockville, MD, USA). More than 25, 000 lncRNAs were collected from the authoritative data sources including NCBI RefSeq, UCSC, NRED and RNAdb. About 5 μg of total RNA from each sample of the 5 matched LSCC cancerous and noncancerous tissues was used for labeling and array hybridization as follows: (1) Double-strand cDNA (ds-cDNA) was synthesized by Superscript ds-cDNA synthesis kit (Invitrogen); (2) ds-cDNA was cleaned and labeled in accordance with Nimblegen one-color DNA labeling kit; (3) Microarrays were hybridized by using the NimbleGen Hybridization System, followed by washing with the Nimblegen wash buffer kit; (4) The slides were scanned using the Axon GenePix 4000B microarray scanner (Molecular Devices Corp., Sunnyvale, CA, USA).

### qPCR

Quantitative real-time RT-PCR (qPCR) was performed as described previously [13]. The gene-specific primers were as follows: LncRNA H19 (H19) (forward: 5′-ATCGGTGCCTCAGCGTTCGG-3′; reverse: 5′-CTGTCCTCGCCGTCACACCG-3′); miR-148a-3p (forward: 5′-AGCAGTTCAGTGCACTACAG-3′; reverse: 5′-GCAGGGTCCGAGGTATTC-3′); DNMT1 (forward: 5′-CCATCAGGCATTCTACCA-3′; reverse: 5′-CGTTC TCCTTGTCTTCTCT-3′); GAPDH (forward: 5′-TTCGA CAGTCAGCCGCATCTT-3′; reverse 5′-CCCAATACG ACCAAATCCGTT-3′). The relative H19 and miR-148a-3p expression levels were calculated using the 2^−DDCt^ (delta Ct) method, with the Ct values normalized using 18S rRNA as internal control. For DNMT1 expression, GAPDH was used as the endogenous control.

### Cell culture and oligonucleotides transfection

Hep-2 cells of human LSCC were cultured in Dulbecco's modified Eagle's medium containing 10% fetal bovine serum (Gibco, Carlsbad, CA, USA) in a humidified incubator (37°C, 5% CO_2_). Isolation and culture of primary laryngeal squamous cell cancer stem cells (LSCC-SCs) was performed as described previously [40]. Hep-2 cells were plated in 24-well plates (2 × 10^4^ cells/well) and cultured overnight. The human H19 shRNA lentiviral transfer vector harboring green fluorescent protein (GFP) and the control lentiviral vector (GFP-lentivirus) were constructed by Genechem (Shanghai, China). The sequences of H19 siRNA are 5′-CCCACAACAUGAAAGAAACTT-3′ (sense) and 5′-AUUUCUUUCAUGU UGUGGGTT-3′ (antisense). The lentiviruses were diluted in 0.2 ml (10^7^ transduction units (TU)/ml) complete medium containing hexadimethrine bromide (Polybrene; 8 mg/ml) and were incubated with the cells for 1 h at 37°C. Next, the cells were incubated with 0.3 ml freshly prepared Polybrene-Dulbecco's modified Eagle's medium for another 24 h; the medium was then replaced with fresh Dulbecco's modified Eagle's medium and the cells were cultured for 48 h. For lentiviral vector transfection, we used Lipofectamine^®^ 2000 Reagent (Invitrogen) according to manufacturer's instruction. For transient transfection, H19 siRNA, control siRNA, miR-148a-3p, miR-148a-3p-in and control miRNA were transfected into the cells using Lipofectamine^®^ RNAiMAX Reagent (Invitrogen) following manufacturer's protocol. Briefly, LSCC cells (5 × 10^5^ per well) were seed into 6-well plate and grown overnight. Lipofectamine^®^ 2000 Reagent (10 μl per well) and DNA (2.5 μg per well) or RNAiMAX reagent (9 μl per well) and oligonucleotides (30 pmol per well) were diluted in 150 μl Opti-MEM serum-free medium respectively, and then mixed together for 5 min at room temperature. The mixture was directly added into each well. After two days transfection, these cells were subjected to the following experiments.

### Wound healing assay

Briefly, cells (2 × 10^5^ per well) were seeded into 12-well plate and grown until 80–90% confluent. The cellular layer was wounded using a 200 μl sterilized tip. After being washed 2 times in PBS, the cells were incubated in growing medium. The wounds at 0 and 36 h were captured by a microscope (Olympus). Migration ability was assessed by measuring changes in sizes of wounded distance.

### Transwell assay

Transwell filters (pore size, 8 μm; Falcon; BD Biosciences, Franklin Lakes, NJ, USA) were coated with 8 μg/μl Matrigel (BD Biosciences) and placed on a 24-well plate containing DMEM. Hep-2 cells (1 × 10^5^) were added to the upper compartment of a transwell chamber and allowed to migrate for 24 h at 37°C. After 24 h, Matrigel and cells remaining on the upper side of the membrane were wiped off with PBS-rinsed cotton swabs. The migrated cells on the lower membrane were stained with calcein acetoxymethyl ester (4 μg/ml, BD Biosciences) in PBS for 30 min, and were observed by fluorescence microscopy (Olympus, Tokyo, Japan). Data were presented as the average number of cells per insert.

### MTS assay

Hep-2 cells were plated at a density of 2, 000 cells/well in 96-well plates. 20 μl MTS (Promega, Madison, WI, USA) was added into the medium after cells were cultured for 0, 1, 2 and 3 days. The cells were then incubated at 37°C with 5% CO_2_ for 2 h, protected from light. Absorbance was detected at 490 nm with a microplate reader (BioRad, Richmond, CA, USA).

### Animal experiments

Sixteen BALB/c mice, age 5 to 6 weeks, were provided by Vital River Laboratories (Beijing, China). They were bred in aseptic conditions and kept at a constant humidity and temperature according to standard guidelines under a protocol approved by Harbin Medical University. All mice were injected subcutaneously in the dorsal scapula region with 100 μl suspension (1 × 10^6^) of Hep-2 cells. The size of the tumor was measured twice a week with calipers, and the volume of tumor was determined using the simplified formula of a rotational ellipsoid (length × width^2^ × 0.5). Once tumors reached approximately 0.5 to 0.6 cm^3^, virus was injected into the tumor once a week for the 3 weeks. Mice in the experimental group (*n* = 8) were treated with 100 μl H19 siRNA lentivirus; mice in the control group (*n* = 8) received an injection of 100 μl GFP lentivirus. Tumors were harvested 1 week after the end of treatment.

### Immunohistochemistry (IHC)

IHC were performed as previously described [41]. Anti-DNMT1 primary antibodies used in IHC were purchased from Santa Cruz Biotechnology (Santa Cruz, CA, USA). Anti-BrdU was purchased from Invitrogen.

### Western blot analysis

Hep-2 cells and LSCC tumors in mice were collected and analyzed using Western blot to assess DNMT1 expression, as described previous [42]. Antibody against DNMT1 and GAPDH were obtained from Santa Cruz Biotechnology. DNMT1 antibody was diluted to 1:1000. GAPDH was used as a loading control on the same membrane.

### MeDIP and microarray hybridization

The methylation status of global DNA of Hep-2 cells transfected with H19 siRNA and control cells was determined by MeDIP-chip using the MeDIP-chip kit, according to the Nimblegen MeDIP-chip protocol. Briefly, genomic DNA was extracted from individual tissue samples and sonicated into 100–500 bp of random fragments. After heat-denaturation, individual DNA samples were probed with 5 μg of mouse anti-5-methylcytidine monoclonal antibody (Eurogenetec, San Diego, CA, USA) in IP buffer (0.5% NP40, 1.1% Triton X-100, 1.5 mM EDTA, 50 mM Tris-HCl, and 150 mM NaCl) with gentle rotation at 4°C overnight. Subsequently, the mixture of DNA and anti-5-methylcytidine was reacted with sheep anti-mouse IgG-conjugated magnetic beads (Bangs laboratories, Fishers, Indiana, USA) at 4°C for 2 h. After washing, the beads were resuspended and the bound proteins were digested with 80 μg of proteinase-K in digestion buffer (50 mM Tris, pH 8.0, 10 mM EDTA, 0.5% SDS) at 50°C for 3 h. The remaining DNA was extracted with phenol-chloroform and precipitated with ethanol. The precipitated DNA was re-suspended in 20 μl of 10 mM Tris-HCl pH 8.0 and used in qPCR for the validation of IP efficiency and for microarray hybridization. The immunoprecipitated methylated DNA was labeled with Cy5 fluorophore and the input genomic DNA was labeled with Cy3 fluorophore. The labeled DNA samples were combined and hybridized to HG18 CpG promoter microarray 385 K (Nimblegen Systems, Inc., Madison, WI, USA). After washing, the arrays were scanned using a GenePix 4000B scanner (Nimblegen Systems, Inc.). Data were extracted and exported to excel using GenePix Pro6.0.

### Luciferase report assay

The pGL3-DNMT1 vector was constructed by amplifying the 3′-UTR of DNMT1 gene harboring the miR-148a-3p binding site predicted by the TargetScan (http://www.targetscan.org) and subsequently cloning it into the pGL3 control vector (Promega) at the Xbal site immediately downstream of firefly luciferase (f-luc). The pGL3-DNMT1-mut vector, which has three mismatch mutations in the miR-148a-3p seed complementary site, was generated to be a negative control. For the luciferase assay, HEK293T cells were cultured in 96-well plates and each cotransfected with 400 ng of either pGL3 DNMT1 or pGL3-DNMT1-mut, 50 ng of pRL-TK Renilla (Promega) and 50 nmol/L miR-148a-3p, miR-148a-3p-in or control miRNA. The pRL-TK Renilla luciferase plasmid was used as an internal control to correct for differences in both transfection and harvest efficiencies. After transfection for 48 h, firefly and Renilla luciferase activities were measured using the dual-luciferase reporter assay (Promega). The results were expressed as relative luciferase activity (firefly luciferase/Renilla luciferase).

### Methylation-specific PCR (MSP) analysis

MSP was performed as previously [43]. Briefly, Genomic DNA was isolated from Hep-2 cells and pre-cleaned. The primers were used for unmethylated MGMT promoter as follows: forward: 5′-GGTCGTTT GTACGTTCGC-3′; reverse: 5′-GACCGATACAAAC CGAACG-3′. For methylation primers: forward: 5′-GT AGGTTGTTTGTATGTTTGT-3′; reverse: 5′-AACCAATA CAAACCAAACA-3′.

### Statistical analysis

All quantitative data were presented as mean ± SEM as indicated from at least three independent experiments. Significance was determined by Student's *t* test or one way ANOVA for group differences. *P* < 0.05 was considered as statistically significant.
